# HIT-Heparin Induced Thrombocytopenia Simulation Case

**DOI:** 10.21980/J89Q0M

**Published:** 2020-01-15

**Authors:** Shaza Aouthmany, Alyssa Siano, De ante’ Russ, Mark Bustillo

**Affiliations:** * University of Toledo College of Medicine, Department of Emergency Medicine, Toledo, OH

## Abstract

**Audience:**

The aim of this simulation is to educate emergency medicine interns, residents and advanced providers on the recognition, diagnosis, and management of heparin-induced thrombocytopenia (HIT). Communication, teamwork, and crisis resource management are incorporated into the case.

**Introduction:**

Heparin-induced thrombocytopenia is a life-threatening complication of heparin exposure (eg, unfractionated heparin, low molecular weight [LMW] heparin) that occurs in a small percentage of patients exposed.^1^ It results in a consumptive coagulopathy in which the clotting cascade is inappropriately activated, leading to a low platelet count yet hypercoagulable state. HIT is associated with mortality rates of 20–30%, with a similar percentage of patients becoming subsequently disabled due to severe complications including limb amputation, multi-system organ failure, stroke or other causes of morbidity.^2^ Heparin-induced thrombocytopenia often presents 5 to 15 days after starting heparin treatment, and with more outpatients being treated with heparin products, it is likely that a first-line provider, such as an emergency medicine physician, would be the first clinician to encounter these patients. The combination of thrombocytopenia and hypercoagulability, along with the rarity of the disease, can make this difficult to diagnose and puts the first line clinician at risk for mismanagement. It is important to be familiar with the signs and symptoms of HIT and to refrain from starting heparin or platelets upon discovery of thrombosis or thrombocytopenia because it will lead to severe and rapid patient deterioration. Therefore, the ability to quickly recognize and treat this condition properly is an essential skill for emergency medicine physicians. Our goal is to create a simulated case that familiarizes emergency providers to this condition so they can be comfortable diagnosing and managing it in a real clinical scenario.

**Educational Objectives:**

After completing this simulated case, participants will be able to:

1. Obtain a detailed history that includes recent medications, medical, surgical, and social history to evaluate for HIT risk factors.
2. Perform an adequate neurovascular exam including evaluation of motor function, sensation, skin color, pulses, and capillary refill.
3. Order appropriate laboratory testing and imaging for diagnosis of thrombocytopenia and arterial occlusion, including bed side doppler or ultrasound.
4. Discuss and recognize the symptoms of HIT and the contraindications of platelet and heparin administration in the emergency department.
5. Avoid administration of heparin in the emergency department setting and recognize that platelets may worsen thrombus formation and lead to limb amputation.^2^
6. Select appropriate medications for treatment and determine appropriate disposition for a patient presenting with HIT.
7. Demonstrate interpersonal communication with patient and family.
8. Recognize that HIT with thrombosis is a potential complication in hospitalized patients and outpatient settings and is associated with high mortality rates.

**Educational Methods:**

This is a high-fidelity simulation case that allows participants to diagnose and treat HIT in a safe environment. The case is followed by a debriefing and small group discussion to review patient care skills, medical knowledge, interpersonal communication, and practice-based learning and improvement.

**Research Methods:**

The educational content and efficacy were evaluated by oral feedback and a debriefing session immediately after completion of the simulation. Participants were provided with an evaluation at the completion of the debriefing session to provide qualitative feedback on the simulation case. A quality Likert Scale was used for the evaluation.

**Results:**

Post-simulation feedback resulted in positive reception, and learners found it useful to run through a high-risk case potentially seen in the emergency department. Out of the 21 participants, 14 responded to the evaluation. Feedback was overwhelmingly positive with the majority rating the simulation as excellent or good.

**Discussion**

The simulation improved awareness of the symptoms of HIT and contraindications of immediate platelet and/or heparin initiation upon vessel occlusion and thrombocytopenia discovery.

**Topics**

Heparin-induced thrombocytopenia, pain control, resuscitation efforts, coagulation disorders, history taking, undifferentiated patient, arterial occlusion, hematology, emergency medicine simulation.

## USER GUIDE

**Table:** 

List of Resources:	
Abstract	24
User Guide	26
Instructor Materials	27
Operator Materials	37
Debriefing and Evaluation Pearls	39
Simulation Assessment	41

**Learner Audience:**

Interns, junior residents, senior residents

**Time Required for Implementation:**

Instructor Preparation: 20–30 minutes

Time for case: 15–20 minutes

Time for debriefing: 10–20 minutes

**Recommended Number of Learners per Instructor:**

2–5

**Topics:**

Heparin-induced thrombocytopenia, pain control, resuscitation efforts, coagulation disorders, history taking, undifferentiated patient, arterial occlusion, hematology, emergency medicine simulation.

**Objectives:**

By the end of this simulation case and debriefing session, the learner will be able to:

1. Obtain a detailed history that includes recent medications, medical, surgical, and social history to evaluate for HIT risk factors.
2. Perform an adequate neurovascular exam including evaluation of motor function, sensation, skin color, pulses, and capillary refill.
3. Order appropriate laboratory testing and imaging for diagnosis of thrombocytopenia and arterial occlusion, including bed side doppler or ultrasound.
4. Discuss and recognize the symptoms of HIT and the contraindications of platelet and heparin administration in the emergency department.
5. Avoid administration of heparin in the emergency department setting and recognize that platelets may worsen thrombus formation and lead to limb amputation.^2^
6. Select appropriate medications for treatment and determine appropriate disposition for a patient presenting with HIT.
7. Demonstrate interpersonal communication with patient and family.
8. Recognize that HIT with thrombosis is a potential complication in hospitalized patients and outpatient settings and is associated with high mortality rates.

### Linked objectives and methods

It is important for emergency medicine physicians to use this high-fidelity simulation method to quickly diagnose HIT and provide an appropriate treatment plan because HIT is associated with high morbidity and mortality (objective 8).^1^,^2^ Learners will need to take a comprehensive history to identify risk factors for HIT as well as perform a thorough neurovascular exam. At this time learners should also order labs and perform a bedside doppler/ultrasound. Upon lab and imaging results learners should discover an arterial occlusion and thrombocytopenia. If learners decide to prematurely treat patient for thrombocytopenia and initiate platelets, they should recognize onset of symptoms and worsening condition of patient (objective 4). However, if the learners demonstrate efficient interpersonal communication with patient and family, they will learn she was treated for DVT (deep vein thrombosis)/PE (pulmonary embolism) as outpatient through her hematologist and has been on unfractionated heparin daily (objective 5,7). This information will lead learners to properly diagnose HIT and disposition the patient accordingly (objective 6).

### Recommended pre-reading for instructor

We recommend any of the reference in the references section.

### Results and tips for successful implementation

This case was written for a high-fidelity simulation scenario and allows learners to diagnose and treat a patient presenting with HIT. This simulation was implemented to be run on emergency medicine interns and residents. The learners were assessed throughout the simulation and given graded percentages on related competencies in the debriefing session. It was noticed that learners were more successful during the simulation when there was optimal teamwork from all participants. The overall feedback from learners and instructors was positive. Out of the 21 residents who participated in this simulation, 14 emergency medicine residents responded and gave feedback. Overall, there was a positive response on this simulation case. Using the Likert scale (1: not a great case to 5: excellent case), 50% of the residents gave the scenario a 5 (excellent), 42.9% rated the case as 4 (Good), and the other 7.1% rated the case as N/A.

## Supplementary Information



## Figures and Tables

**Table:** 

#1	Complete blood count (CBC)
#2	Basic metabolic panel (BMP)
#3	Prothrombin time and international normalized ratio (PT/INR)
#4	Activated partial thromboplastin time (aPTT)
#5	Fibrinogen
#6	Troponin
#7	Electrocardiogram (ECG)
#8	Computed tomography angiography (CTA)
#9	Bedside doppler/ultrasound

**Table:** 

1.	Place patient on the monitor
2.	Obtain full history from the patient
3.	Obtain bedside imaging and order proper labs
4.	Discover thrombocytopenia
5.	Do not immediately transfuse platelets
6.	Discover arterial occlusion
7.	Establish diagnosis of heparin induced thrombocytopenia
8.	Obtain vascular and hematology consults
9.	Administer argatroban and/or lepirudin
10.	Admit patient to intermediate/telemetry care

**Table:** 

**Vitals:**	*Heart Rate (HR)* 111	*Blood Pressure (BP)* 170/106	*Respiratory Rate (RR)* 20
	*Temperature (T)* 37°C	*Oxygen Saturation (O_2_Sat)* 100% on room air	

**Table:** 

*Complete blood count (CBC)*	
White blood count (WBC)	4.4 x1000/mm^3^(H)
Hemoglobin (Hgb)	12.0 g/dL
Hematocrit (HCT)	37.0%
Platelet (Plt)	33 x1000/mm^3^

**Table:** 

*Basic metabolic panel (BMP)*	
Sodium	139 mEq/L
Chloride	108 mEq/L
Potassium	4.6 mEq/L
Bicarbonate (HCO_3_)	21 mEq/L (L)
Blood Urea Nitrogen (BUN)	17 mg/dL (H)
Creatine (Cr)	1.1 mg/dL (H)
Glucose	143 mg/dL

**Table:** 

*Prothrombin time (PT)*	1.1 seconds

**Table:** 

*International normalized ratio (INR)*	15

**Table:** 

*Activated Partial Thromboplastin Time* (aPTT)	40 seconds

**Table:** 

*Fibrinogen*	450 mg/dL

**Table:** 

*Troponin*	0.01 mcg/dL

**Figure:**
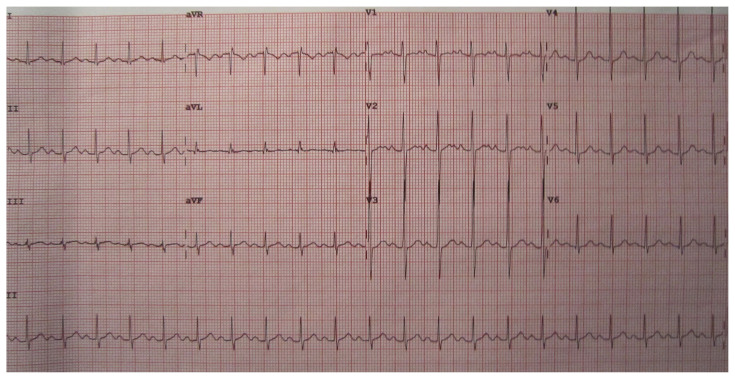
ECG shows sinus tachycardia.

**Figure:**
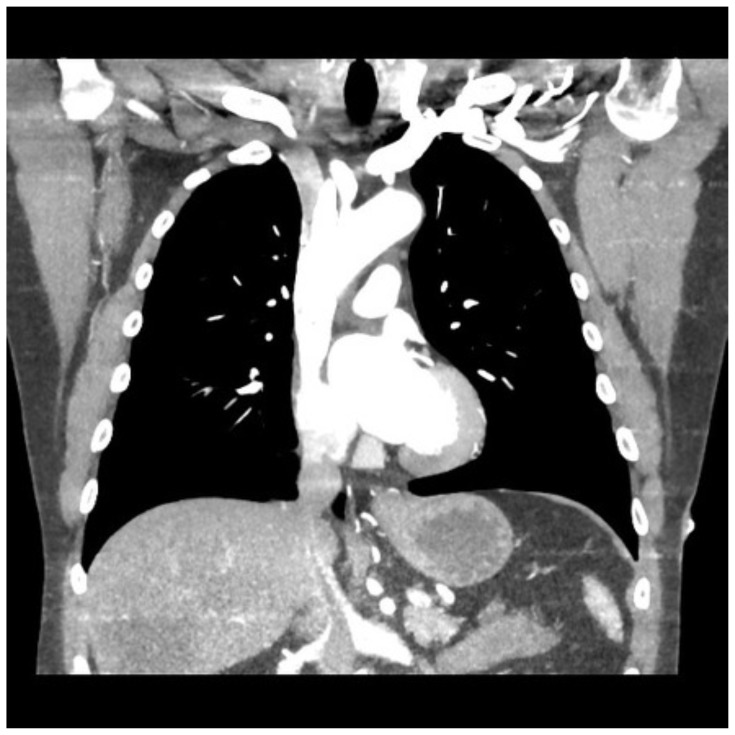
Normal CTA chest

**Figure:**
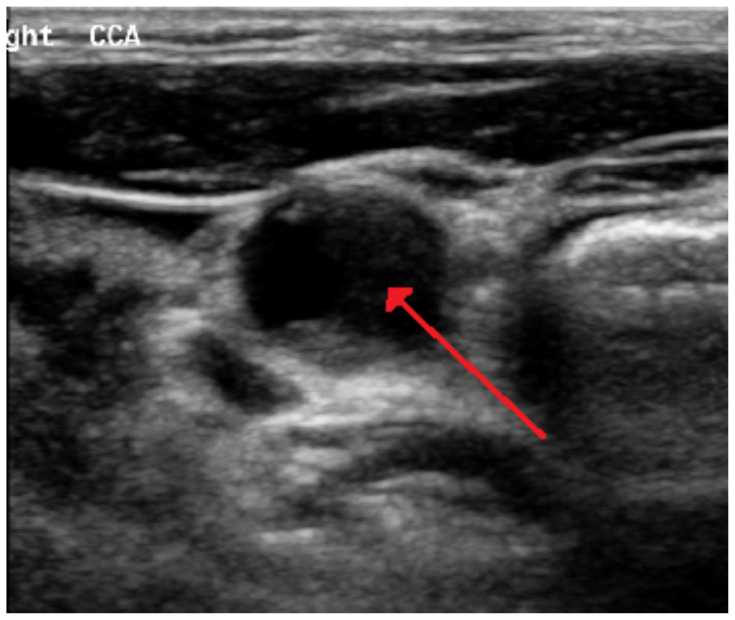
Imaging shows arterial occlusion

**Table:** 

Minute (state)	Participant action/trigger	Patient status (simulator response) & operator prompts	Monitor display (vital signs)
0:00 (Baseline)	-Obtain full history because recent medications will lead to proper diagnosis -Have broad differential for paresthesias -Perform adequate neurovascular exam -Immediately obtain bedside doppler or ultrasound -Treat pain	If learners do not recognize possible arterial occlusion patient continues to complain of worsening pain. -Transition: After history/physical move to state 2 -Labs: Glucose 143 - Breath sounds: Clear -Skin: Flushed -LOC: GCS 15 -Pain: Right hand	T: 37° C HR: 111 BP: 170/106 RR: 20 O2: 100% on room air (RA) ECG (rhythm): Sinus tachycardia
State 2: Post exam	-Do not immediately transfuse platelets upon finding thrombocytopeni a -Patient noted she was here for PE and has been on unfractionated heparin daily, last labs show platelets 175 -Treat pain -Obtain vascular and hematology consult	Patient states she is being treated for PE/DVT and has been on unfractionated heparin daily -Transition: If argatroban ordered and platelets not given, go to state 4 -Transition: If platelets given go to state 3 -Labs: Hgb 12, Plt 33, Trop .01, PT/INR 15/1.1, Apt 40, Fibrinogen 450 -Breath sounds: Clear -Skin: Flushed -LOC: GCS 15 -Pain: Better with medication	HR: 101 BP: 165/101 RR: 20 02: 100% RA ECG (rhythm): Sinus tachycardia
State 3	-Participant should recognize the change after initiation of platelets	Patient decompensates--patient will develop worsening extremity pain, cyanosis in fingertips, shortness of breath, chest pain, and tachycardia. -Breath sounds: Clear -Skin: Flushed -LOC: GCS 15 -Pain: Worsening right hand pain, patient now has calf pain and SOB	T: 38.5° C HR: 130 BP: 200/122 RR: 25 O2: 97% RA ECG (rhythm): Sinus tachycardia
State 4-Case completion	-Recognize patient needs to be admitted -Do not give heparin -Do not give platelets -Start argatroban and lepirudin	-Labs: Anti-PF4 pending, SRA pending -Breath sounds: Clear -Skin: Flushed -LOC: GCS 15 -Pain: Improved	T: N/A HR: 96 BP: 155/89 RR: 20 O2: 100% RA ECG (rhythm): Normal sinus rhythm

**Table:** 

**Critical Actions** Place patient on the monitor Obtain full history from the patient Obtain bedside imaging and order proper labs Discover thrombocytopenia Do not immediately transfuse platelets Discover arterial occlusion Establish diagnosis of heparin induced thrombocytopenia Obtain vascular and hematology consults Administer argatroban and/or lepirudin Admit patient to intermediate/telemetry care	0.00

**Table:** 

	Milestone	Did not achieve level 1	Level 1	Level 2	Level 3
1	**Emergency Stabilization (PC1)**	□ Did not achieve Level 1	□ Recognizes abnormal vital signs	□ Recognizes an unstable patient, requiring intervention Performs primary assessment Discerns data to formulate a diagnostic impression/plan	□ Manages and prioritizes critical actions in a critically ill patient Reassesses after implementing a stabilizing intervention
2	**Performance of focused history and physical (PC2)**	□ Did not achieve Level 1	□ Performs a reliable, comprehensive history and physical exam	□ Performs and communicates a focused history and physical exam based on chief complaint and urgent issues	□ Prioritizes essential components of history and physical exam given dynamic circumstances
3	**Diagnostic studies (PC3)**	□ Did not achieve Level 1	□ Determines the necessity of diagnostic studies	□ Orders appropriate diagnostic studies. Performs appropriate bedside diagnostic studies/procedures	□ Prioritizes essential testing Interprets results of diagnostic studies Reviews risks, benefits, contraindications, and alternatives to a diagnostic study or procedure
4	**Diagnosis (PC4)**	□ Did not achieve Level 1	□ Considers a list of potential diagnoses	□ Considers an appropriate list of potential diagnosis May or may not make correct diagnosis	□ Makes the appropriate diagnosis Considers other potential diagnoses, avoiding premature closure
5	**Pharmacotherapy (PC5)**	□ Did not achieve Level 1	□ Asks patient for drug allergies	□ Selects an medication for therapeutic intervention, consider potential adverse effects	□ Selects the most appropriate medication and understands mechanism of action, effect, and potential side effects Considers and recognizes drug-drug interactions
6	**Observation and reassessment (PC6)**	□ Did not achieve Level 1	□ Reevaluates patient at least one time during case	□ Reevaluates patient after most therapeutic interventions	□ Consistently evaluates the effectiveness of therapies at appropriate intervals
7	**Disposition (PC7)**	□ Did not achieve Level 1	□ Appropriately selects whether to admit or discharge the patient	□ Appropriately selects whether to admit or discharge Involves the expertise of some of the appropriate specialists	□ Educates the patient appropriately about their disposition Assigns patient to an appropriate level of care (ICU/Tele/Floor) Involves expertise of all appropriate specialists
9	**General Approach to Procedures (PC9)**	□ Did not achieve Level 1	□ Identifies pertinent anatomy and physiology for a procedure Uses appropriate Universal Precautions	□ Obtains informed consent Knows indications, contraindications, anatomic landmarks, equipment, anesthetic and procedural technique, and potential complications for common ED procedures	□ Determines a back-up strategy if initial attempts are unsuccessful Correctly interprets results of diagnostic procedure
20	**Professional Values (PROF1)**	□ Did not achieve Level 1	□ Demonstrates caring, honest behavior	□ Exhibits compassion, respect, sensitivity and responsiveness	□ Develops alternative care plans when patients’ personal beliefs and decisions preclude standard care
22	**Patient centered communication (ICS1)**	□ Did not achieve level 1	□ Establishes rapport and demonstrates empathy to patient (and family) Listens effectively	□ Elicits patient’s reason for seeking health care	□ Manages patient expectations in a manner that minimizes potential for stress, conflict, and misunderstanding. Effectively communicates with vulnerable populations, (at risk patients and families)
23	**Team management (ICS2)**	□ Did not achieve level 1	□ Recognizes other members of the patient care team during case (nurse, techs)	□ Communicates pertinent information to other healthcare colleagues	□ Communicates a clear, succinct, and appropriate handoff with specialists and other colleagues Communicates effectively with ancillary staff

## INSTRUCTOR MATERIALS

**Case Title:** HIT-Heparin Induced Thrombocytopenia

**Case Description & Diagnosis (short synopsis):** 66-year-old female Patty Myers presents to the emergency department (ED) with localized right-hand tingling and pain. She states it started that morning. She denies any trauma. Patient’s child is angry that the patient is back in the hospital. The patient gives a history and states she was recently treated for a DVT/PE as outpatient. The patient’s pain will continue to increase until learners recognize the diagnosis and start appropriate treatment.

**Equipment or Props Needed:**

High-fidelity adult simulator or simulated patient actor
Imaging results (doppler or ultrasound, CTA of chest)
Lab results
Electrocardiogram (ECG)
IV pole
Crash cart with defibrillator
Simulation events table, simulation assessment timeline, simulation assessment and milestones

**Confederates needed:**

Patient’s child pharmacist, vascular and hematology physicians for consultation (all voiced by operator)

**Stimulus Inventory:**

**Background and brief information:** The case scenario occurs in the emergency department at a hospital with level of care and resources determined by the operator. The patient is a 66-year-old female who arrives as a walk-in and is alert, awake and oriented at time of arrival. She complains of localized right-hand pain and tingling.

**Initial presentation:** The patient presents in the emergency department with right hand pain, decreased sensation, and tingling. Patient’s skin is flushed, there is edema present, and baseline vital signs show elevated heart rate and blood pressure. Additionally, there are no lacerations, patient has full range of motion, and poor capillary refill.

**How the scenario unfolds:**

The patient presents to the emergency department with localized right-hand pain and tingling. The participants should place patient on the monitor and obtain a full set of vital signs as they gain history from patient and family. Patient denies any trauma, provides some of her history, and states she was recently treated for a DVT (Deep Vein Thrombosis) or pulmonary embolism (PE) seven days ago by her hematologist. She explains that her pain started 12 hours ago. It was also noted that patient was on unfractionated heparin daily. The learners should perform a complete neurovascular exam signaling to the learner there is a vascular problem. Additionally, laboratory and bedside imaging (doppler/ultrasound) may be requested at this time. It is likely the learner may order an alternative modality of imaging to evaluate extremity vasculature that may include angiography or computed tomography with contrast. In the case of the learner ordering a different modality of imaging, the learner should be informed that this imaging is not available in this hospital setting or the CT is not currently functioning. Ideally, the learner should come to the conclusion that bedside ultrasound is the imaging of choice to evaluate the patient’s findings. The arterial occlusion and thrombocytopenia should then be discovered after appropriate orders have been placed. If learner does not recognize possible arterial occlusion, the patient continues to complain of worsening pain. If the learner continues to fail to make the appropriate diagnosis of arterial occlusion, the patient’s symptoms may evolve to more classic signs of arterial occlusion that can include pain out of proportion, pallor, paralysis, pulselessness or cold extremity. Upon discovery of thrombocytopenia, platelets should not be immediately transfused. If learners do not properly diagnose patient with HIT and platelets or heparin is given, the patient begins to decompensate. Learners should recognize elevation in blood pressure, respiratory rate, and onset of shortness of breath following initiation of platelets. Additionally, patient’s pain in right hand will worsen and pain will begin in calf. If learners do properly diagnose patient with HIT, they should recognize the need for admission, obtain vascular and hematology consults, and initiate argatroban and/or lepirudin. If the operator believes the case should be more challenging, they can provide the learner with a “poor historian” patient.

**Critical Actions:**

**Case Title:** HIT-Heparin Induced Thrombocytopenia

**Chief Complaint:** 66-year-old female complaining of right-hand pain and tingling that started the same morning.

**General Appearance:** 66-year-old female, appears stated age, in moderate distress

**Primary Survey:**

- **Airway:** Clear
- **Breathing:** Patient’s breath sounds are clear, RR and Sp02 are within normal range
- **Circulation:** Poor capillary refill in affected hand, normal capillary refill in other extremities, tachycardic

**History:**

- **History of present illness**: 66-year-old patient presents to the ED with localized right-hand tingling and pain that started this morning. Patient denies any trauma. When prompted she states that she was treated for DVT/PE seven days ago. It was also noted she was on unfractionated heparin daily.
- **Past medical history:** Diabetes mellitus, hypertension, coronary artery disease, HPL, fibromyalgia, depression, bipolar, colon cancer, recent outpatient treatment for DVT/PE
- **Past surgical history:** Right femur fracture
- **Patients medications:** Metformin, ASA, Lipitor, gabapentin, folate, insulin, lisinopril
- **Allergies:** Penicillin
- **Social history:** Patient currently uses smokeless tobacco and occasionally uses cocaine in social settings.
- **Family history:** Diabetes mellitus, hypertension, cerebrovascular accident, pulmonary embolism

**Secondary Survey/Physical Examination:**

- **General appearance:** Moderate distress
- **Head, ears, eyes, nose and throat (HEENT):**
  ○ **Head:** Within normal limits
  ○ **Eyes:** Within normal limits
  ○ **Ears:** Within normal limits
  ○ **Nose:** Within normal limits
  ○ **Throat:** Within normal limits
- **Neck:** Within normal limits
- **Heart:** Tachycardic rate, regular rhythm
- **Lungs:** Within normal limits
- **Abdominal/GI:** Within normal limits
- **Genitourinary:** Within normal limits
- **Rectal:** Within normal limits
- **Extremities:** Decreased sensation, moderate pain, swelling, paleness and prolonged capillary refill in right hand, otherwise within normal limits
- **Back:** Within normal limits
- **Neuro:** Within normal limits
- **Skin:** Flushed, edema present
- **Lymph:** Within normal limits
- **Psych:** Within normal limits

**Results:**

*Electrocardiogram (ECG)*

Heilman J. Sinus tachycardia as seen on ECG. In:

Wikimedia.org.commons.wikimedia.org/wiki/file:sinustachy.JPG. Published June 15, 2012. Accessed June 9, 2020. CC BY-SA 3.0.

*Computed Tomography Angiography (CTA) Chest*

Hacking C. Normal CTA Thorax (ECG gated). In: Radiopaedia.org. 18 December 2015. Published

December 18, 2015. Accessed June 9, 2020. CC BY-NC-SA 3.0. radiopaedia.org/images/18195635

*Bedside Doppler/Ultrasound*

Heilman J. 70perblockedrtcarotida. In: Wikimedia.org. 28 May 2016. Published 28 May 2016. Accessed 5 November 2020. CC BY-SA 4.0.

https://commons.wikimedia.org/wiki/File:70PerBlockedRtCarotidA.png

## OPERATOR MATERIALS

**SIMULATION EVENTS TABLE:**

**Diagnosis:** Heparin Induced Thrombocytopenia

**Disposition:** Admit patient appropriately to intermediate/telemetry care

## DEBRIEFING AND EVALUATION PEARLS

### HIT-Heparin Induced Thrombocytopenia

**Background/Causes:**

- Heparin induced thrombocytopenia is a life-threatening immune-mediated adverse drug reaction caused by the production of antibodies that activate platelets in the presence of heparin.^1^ A potential life-threatening complication of HIT is thrombosis that can occur anywhere in the body but commonly causes extremity arterial occlusion, atrial appendage thrombosis, cardiac ischemia and stroke. Thrombosis typically occurs concurrently with thrombocytopenia and contributes to high rates of disease mortality.^1^,^2^

**Clinical Presentation:**

- Acute symptoms include pain, swelling, flushing, hypertension, tachycardia, and dyspnea but can also include more severe signs including skin necrosis or multi-organ system failure.^3^
- Onset of thrombocytopenia within 5–10 days following heparin treatment^1^
- Learners should look for the “4 T’s” of HIT to help identify patients who are at risk which include Thrombocytopenia, Timing, Thrombosis and other causes for Thrombocytopenia.^1^,^3^

**Workup findings:**

- Right hand arterial occlusion
- Thrombocytopenia

**Treatment/management:**

- **Medications:**
  ○ Some alternative anticoagulants available for treatment include argatroban and lepirudin they do not cross-react with HIT antibodies^1^
- **Argatroban:**
  ○ The recommended initial dose is 2μg/kg per minute, regulated to achieve aPTTs 1.5–3 times the baseline value^3^
  ○ Treatment with argatroban has shown no evidence of antibody generation on prolonged or repeated administration and has no reported anaphylactic deaths^1^
- **Lepirudin:**
  ○ The recommended initial dose is 0.4-mg/kg bolus followed by 0.15-mg/kg per hour infusion, regulated to aPTT ratios of 1.5–2.5^3^
  ○ Lepirudin lowers the chances of thromboembolic complications and limb amputation^4^

**Other debriefing points:**

- Discuss evaluation and treatment of HIT
- Discuss the contraindications of immediate heparin/platelet initiation in a patient presenting with HIT
- Discuss benefits and dosing of argatroban/lepirudin

## SIMULATION ASSESSMENT

*HIT-Heparin Induced Thrombocytopenia Simulation Case*

Learner: _________________________________________

***Assessment Timeline***

This timeline is to help observers assess their learners. It allows observer to make notes on when learners performed various tasks, which can help guide debriefing discussion.

**Critical Actions:**

□ Place patient on the monitor
□ Obtain full history from the patient
□ Obtain bedside imaging and order proper labs
□ Discover thrombocytopenia
□ Do not immediately transfuse platelets
□ Discover arterial occlusion
□ Establish diagnosis of heparin induced thrombocytopenia
□ Obtain vascular and hematology consults
□ Administer argatroban and/or lepirudin
□ Admit patient to intermediate/telemetry care

**Summative and formative comments:**

**Milestones assessment:**
