# Supplementary material for: HIT-Heparin Induced Thrombocytopenia Simulation Case
Source: J Educ Teach Emerg Med. 2020 Jan 15;6(1):S24–45. doi: 10.21980/J89Q0M (PMC10332759; doi:10.21980/J89Q0M)
Supplement: Supplementary file 1 [file jetem-6-1-s24-supp1.pptx]

## Slide 1
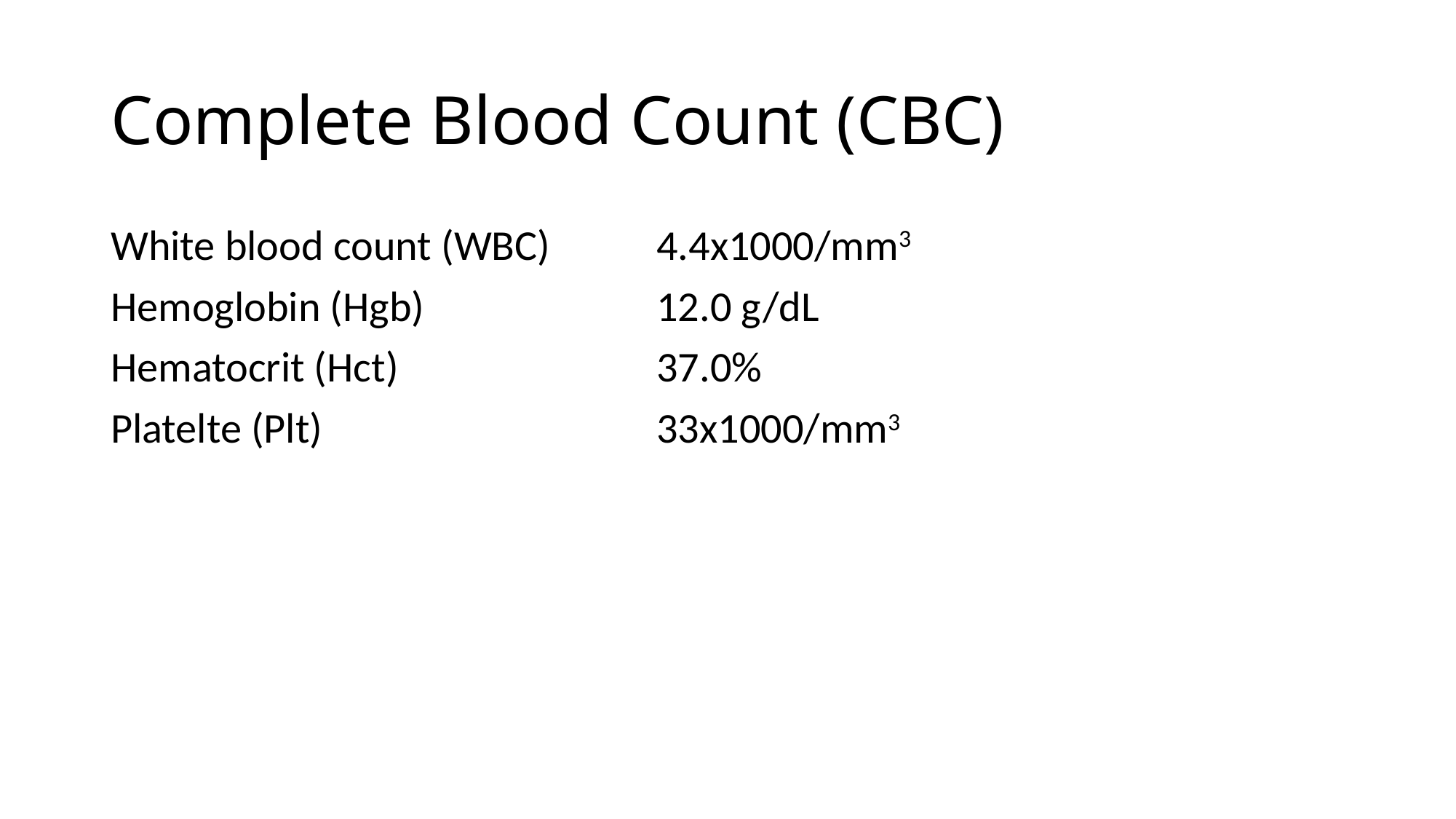

# Complete Blood Count (CBC)
White blood count (WBC)	4.4x1000/mm3
Hemoglobin (Hgb) 			12.0 g/dL
Hematocrit (Hct)			37.0%
Platelte (Plt)				33x1000/mm3

## Slide 2
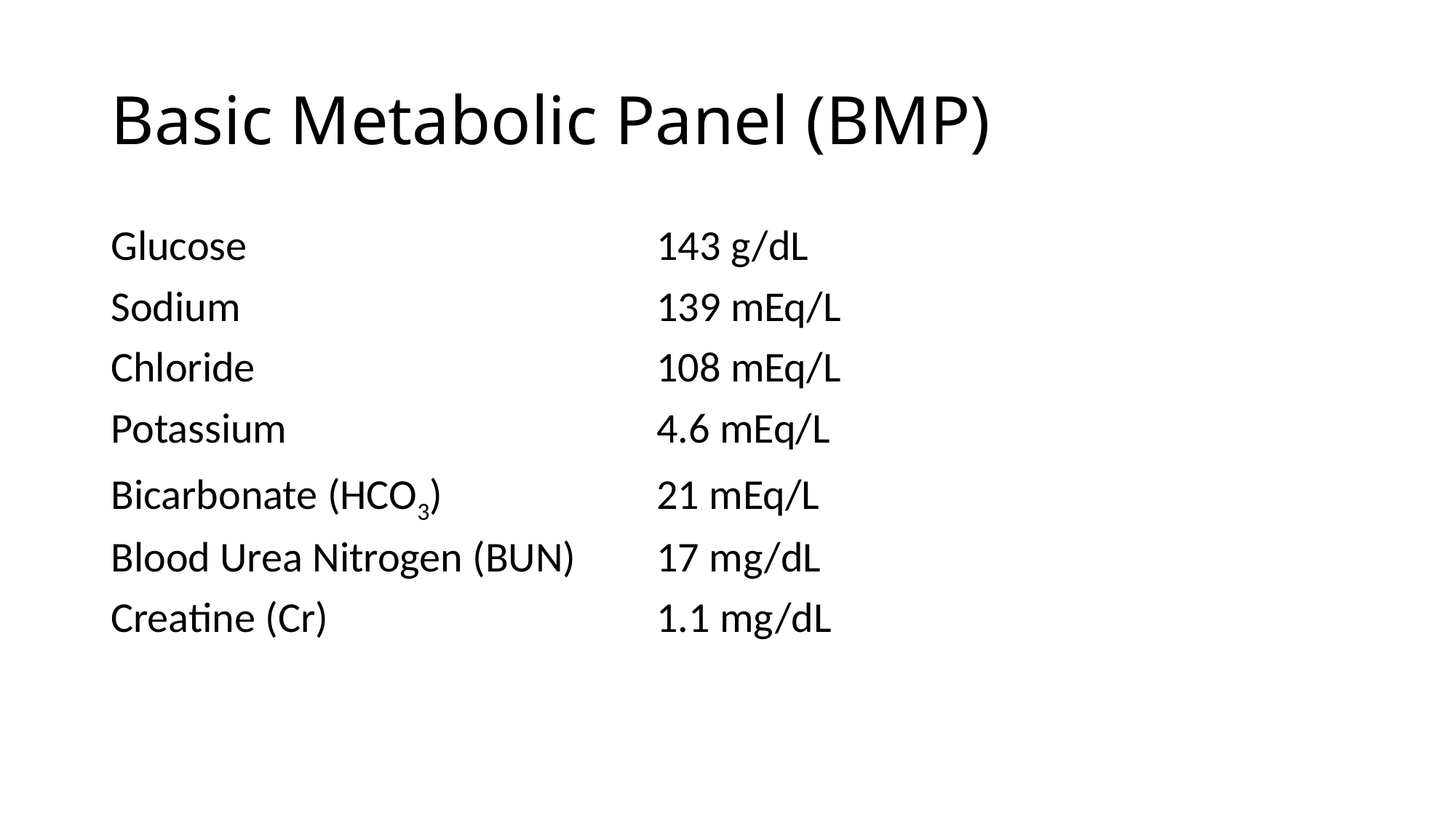

# Basic Metabolic Panel (BMP)
Glucose				143 g/dL
Sodium				139 mEq/L
Chloride				108 mEq/L
Potassium 				4.6 mEq/L
Bicarbonate (HCO3)		21 mEq/L
Blood Urea Nitrogen (BUN)	17 mg/dL
Creatine (Cr)				1.1 mg/dL

## Slide 3
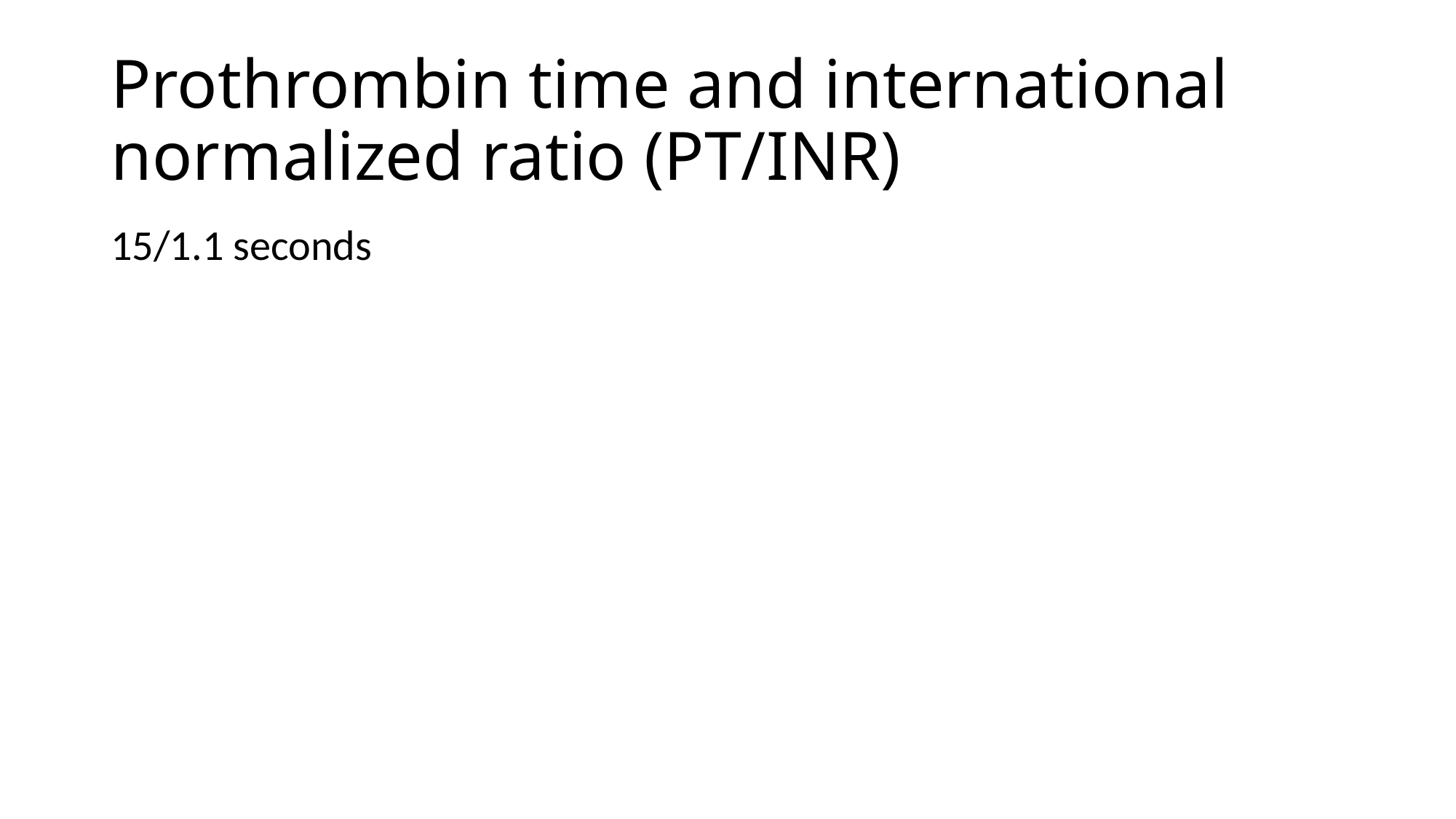

# Prothrombin time and international normalized ratio (PT/INR)
15/1.1 seconds

## Slide 4
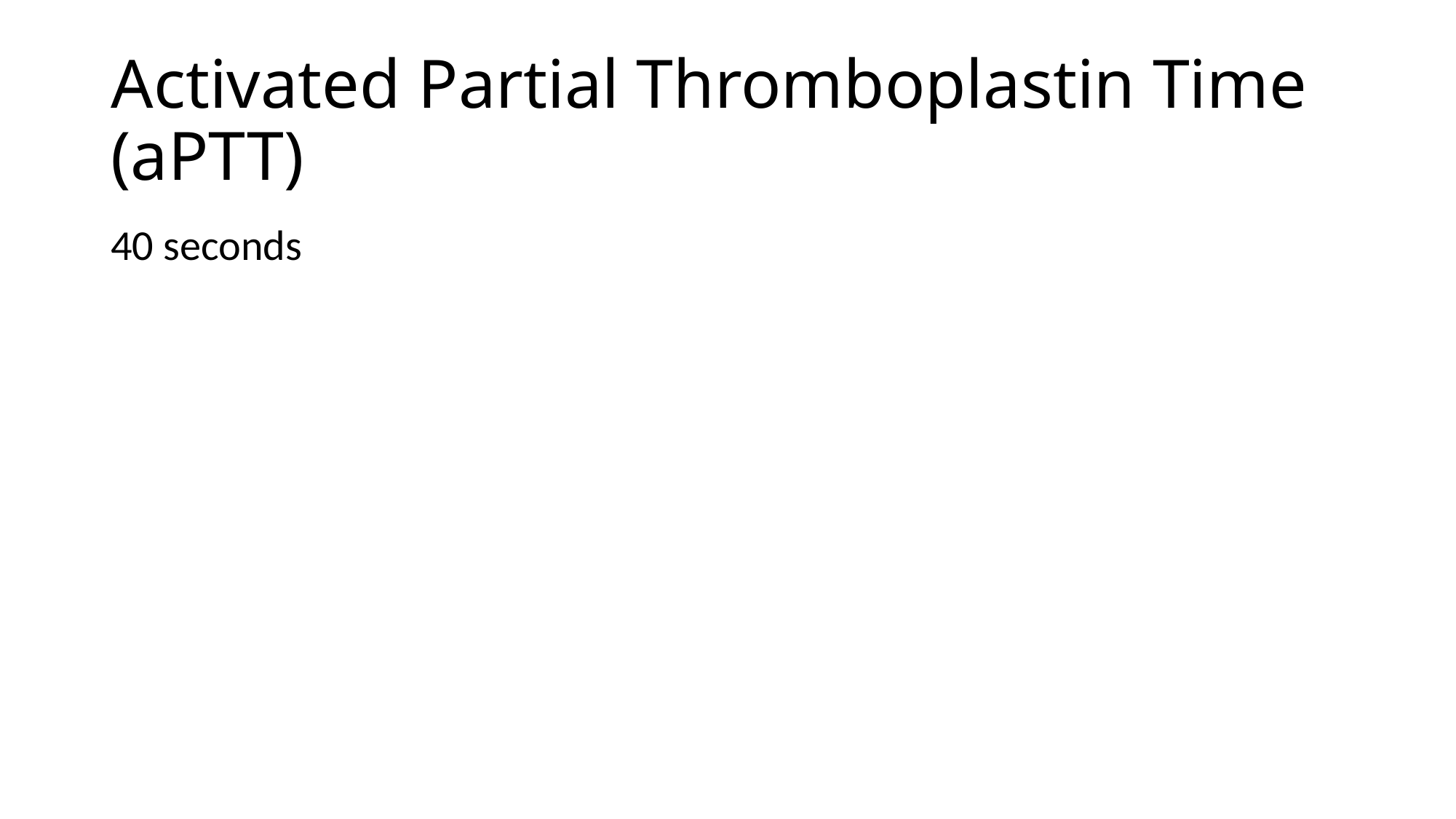

# Activated Partial Thromboplastin Time (aPTT)
40 seconds

## Slide 5
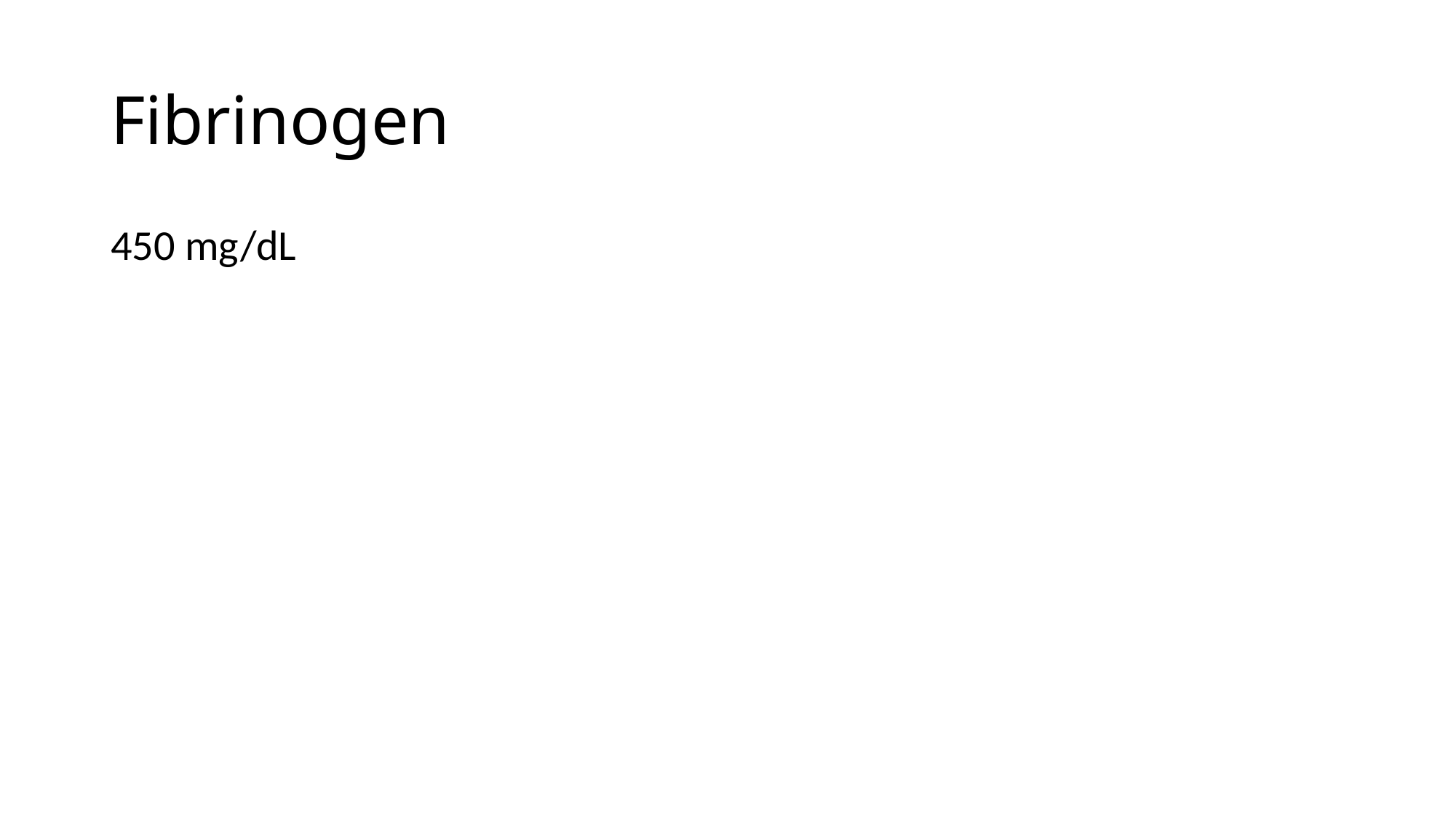

# Fibrinogen
450 mg/dL

## Slide 6
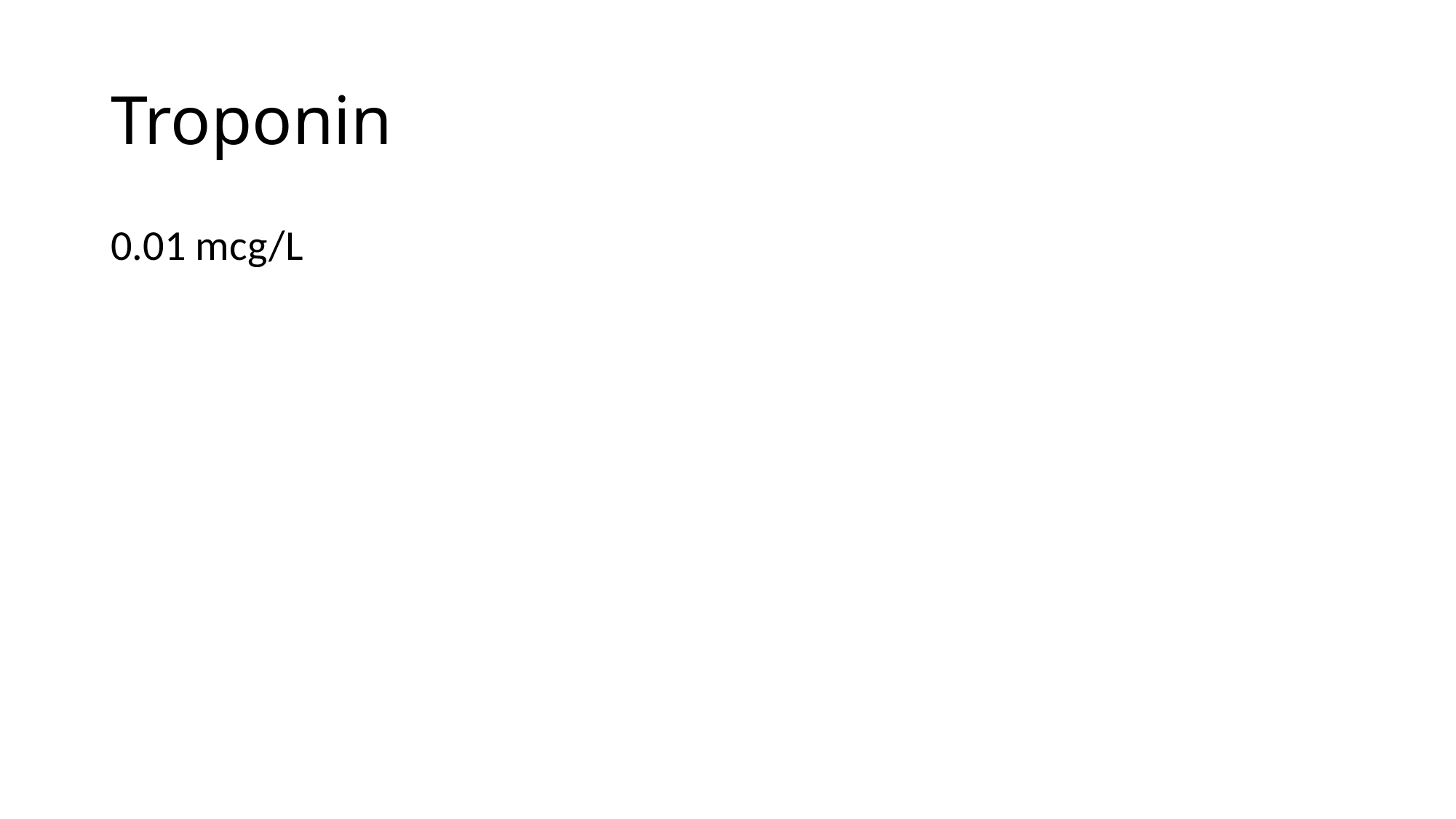

# Troponin
0.01 mcg/L

## Slide 7
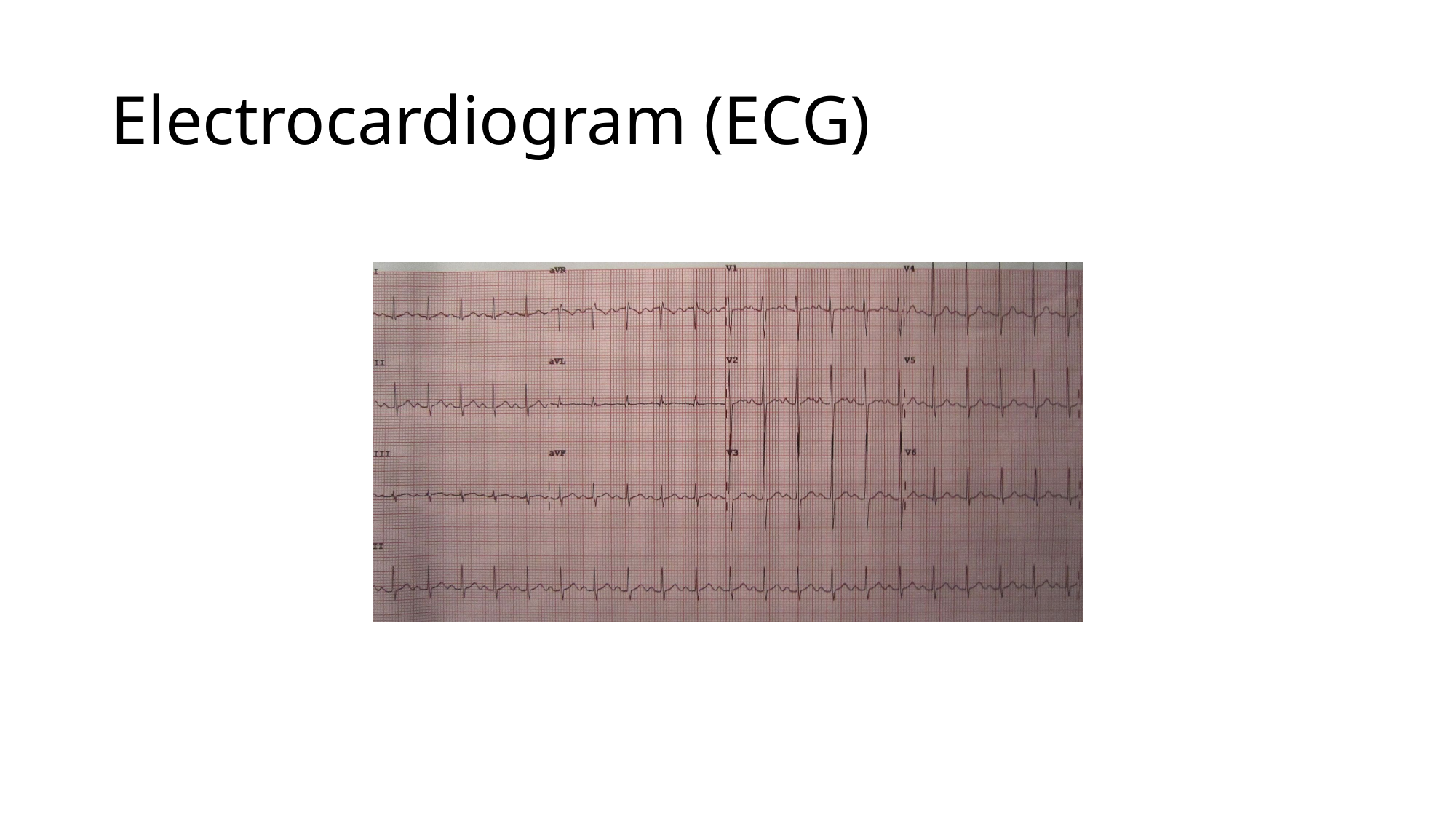

# Electrocardiogram (ECG)

## Slide 8
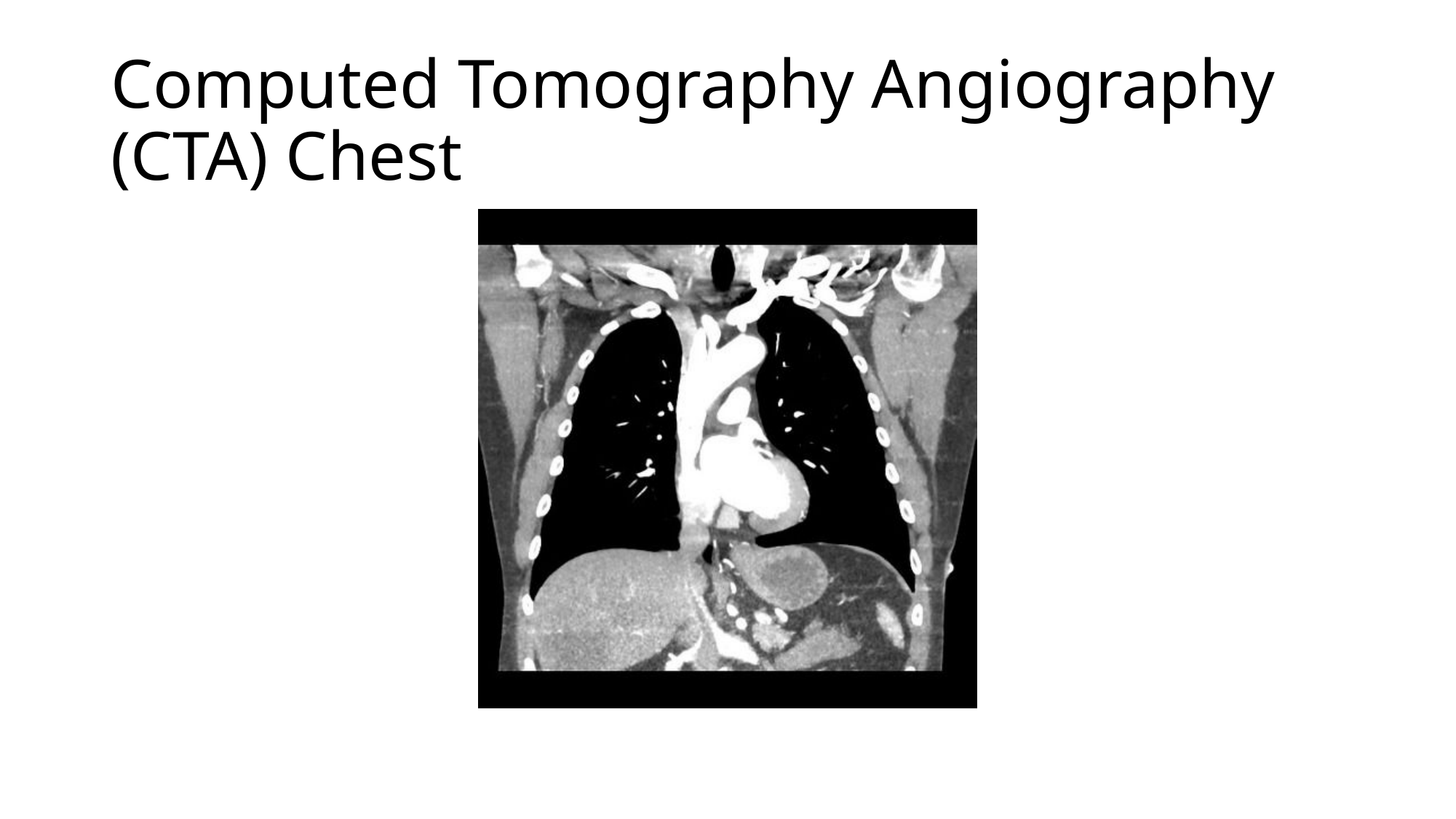

# Computed Tomography Angiography (CTA) Chest

## Slide 9
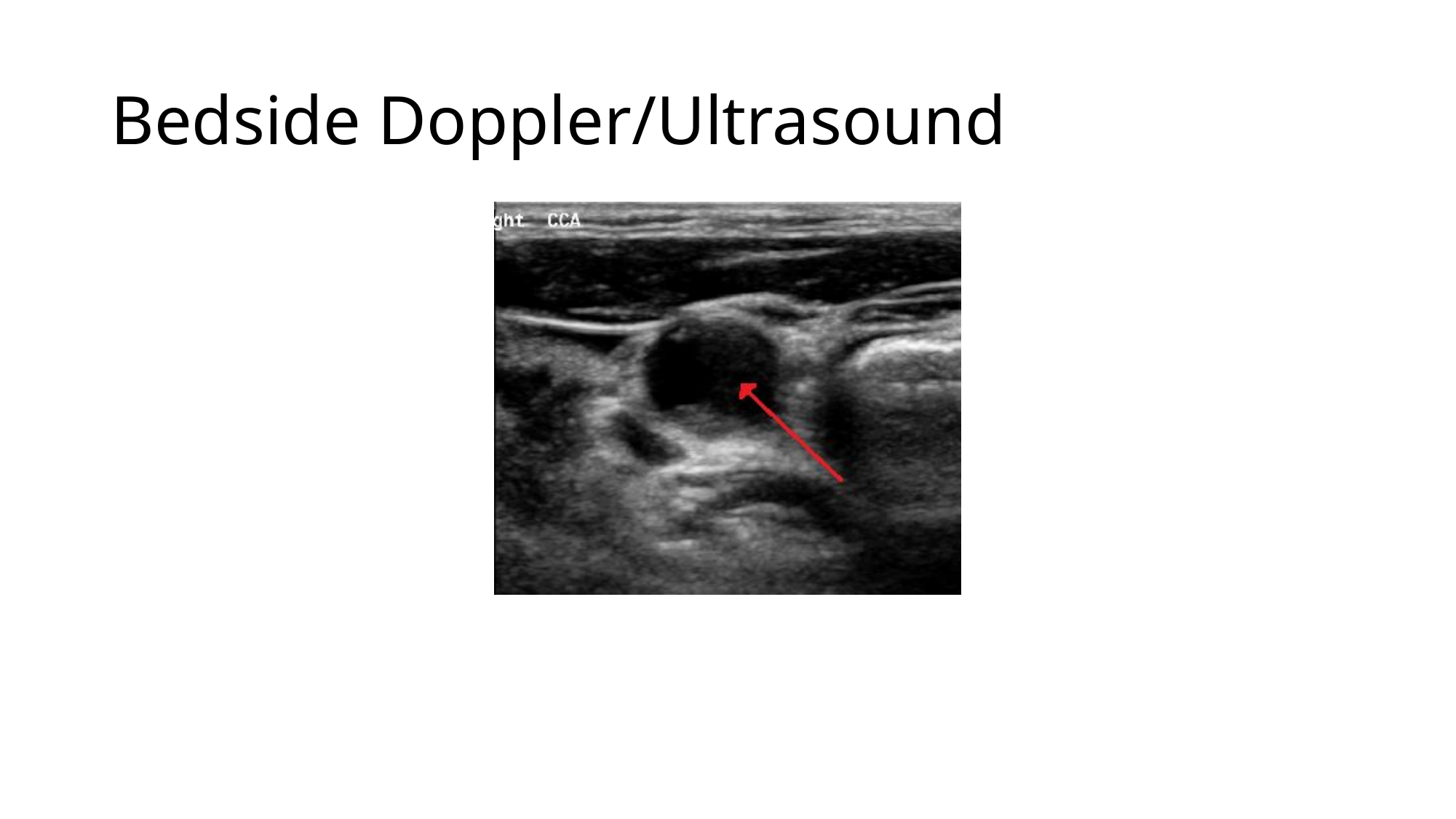

# Bedside Doppler/Ultrasound
